# The protective effect of the PDE-4 inhibitor rolipram on intracerebral haemorrhage is associated with the cAMP/AMPK/SIRT1 pathway

**DOI:** 10.1038/s41598-021-98743-w

**Published:** 2021-10-05

**Authors:** Xiao-Liu Dong, Yan-Hui Wang, Jing Xu, Nan Zhang

**Affiliations:** 1 Department of Neurology, Tianjin Medical University General Hospital, Tianjin, 300052 China; 2 Department of Neurorehabilitation, Tangshan People’s Hospital, Tangshan, 063000 China

**Keywords:** Molecular biology, Neurology

## Abstract

Rolipram specifically inhibits phosphodiesterase (PDE) 4, thereby preventing inactivation of the intracellular second messenger cyclic adenosine monophosphate (cAMP). Rolipram has been shown to play a neuroprotective role in some central nervous system (CNS) diseases. However, the role of PDE4 and the potential protective effect of rolipram on the pathophysiological process of intracerebral haemorrhage (ICH) are still not entirely clear. In this study, a mouse model of ICH was established by the collagenase method. Rolipram reduced brain oedema, blood–brain barrier (BBB) leakage, neuronal apoptosis and inflammatory cytokine release and improved neurological function in our mouse model of ICH. Moreover, rolipram increased the levels of cAMP and silent information regulator 1 (SIRT1) and upregulated the phosphorylation of AMP-activated protein kinase (AMPK). Furthermore, these effects of rolipram could be reversed by the SIRT1 inhibitor sirtinol. In conclusion, rolipram can play a neuroprotective role in the pathological process of ICH by activating the cAMP/AMPK/SIRT1 pathway.

## Introduction

Stroke is an acute cerebral vascular disease^1^ and a common neurological disorder with high rates of recurrence, disability and mortality^2^. Globally, there is a huge burden associated with stroke, with 10.3 million new strokes and 113 million disability adjusted life years (DALYs) per year^3^. Intracerebral haemorrhage (ICH) accounts for approximately 10–15% of all strokes^4^. Although great progress has been made in research focusing on the pathogenesis of ICH, the neurological dysfunction caused by ICH still imposes a heavy burden on patients, their families, and even society^5^. Therefore, it is necessary to develop an effective intervention strategy for patients with ICH.

The pathological mechanism of ICH is very complex and is closely associated with a series of pathophysiological processes in the central nervous system (CNS), such as inflammatory reactions, neuronal apoptosis, blood–brain barrier (BBB) injury and brain oedema^6^. Among them, brain oedema is mainly caused by BBB disruption and is an extremely harmful key factor that leads to neurological impairment during the early stage of ICH^7^. Subsequently, a variety of secondary pathological changes can exacerbate neurological deficits^8,9^.

As an important second messenger inside the cell, cyclic adenosine monophosphate (cAMP) is involved in many physiological functions in humans. Phosphodiesterases (PDEs) are cAMP specific hydrolases^10^ that are responsible for hydrolysing cAMP to the corresponding inactive 5′-phosphate, thereby terminating all cellular activities mediated by cAMP signalling^11^. PDEs are a superfamily of enzymes and include PDE1-11, of which PDE4 is the largest member and is widely expressed in the CNS^12^. At the tissue level, approximately 80% of PDE4 is expressed in brain tissue^12^; at the cellular level, PDE4 is mainly expressed in inflammatory cells^13^ and nerve cells^14^. These distribution characteristics determine the biological functions of PDE4, such as participation in the immune response and nerve regeneration, survival and repair. A recent study showed that the specific PDE4 inhibitor rolipram ameliorated brain oedema and alleviated neurological dysfunction after subarachnoid haemorrhage (SAH)^15^. However, the effects of PDE4 and rolipram on the pathogenesis of ICH are still not fully understood.

Studies have reported that cAMP-dependent pathways can activate AMP-activated protein kinase (AMPK)^16^, which activates niacinamide phosphoribosyltransferase at the transcriptional level and then induces silent information regulator 1 (SIRT1) activation^17^. SIRT1 activation has been demonstrated to participate in the regulation of a series of biological processes, including protection of the BBB, anti-inflammation, and anti-apoptosis^18^. Previous studies have shown that rolipram plays a neuroprotective role by regulating the cAMP/AMPK/SIRT1 signalling pathway to reduce the consumption of ATP during cerebral ischaemia^19^. However, direct evidence about the neuroprotective effect of rolipram on the regulation of the cAMP/AMPK/SIRT1 after ICH is still lacking. Therefore, a mouse model was used to explore the function and mechanism of rolipram in the pathophysiological process of ICH to provide evidence for this new potential therapeutic agent to treat patients with ICH.

## Results

### The distribution of PDE4 in the surgical hemisphere after ICH

**Figure 1:**
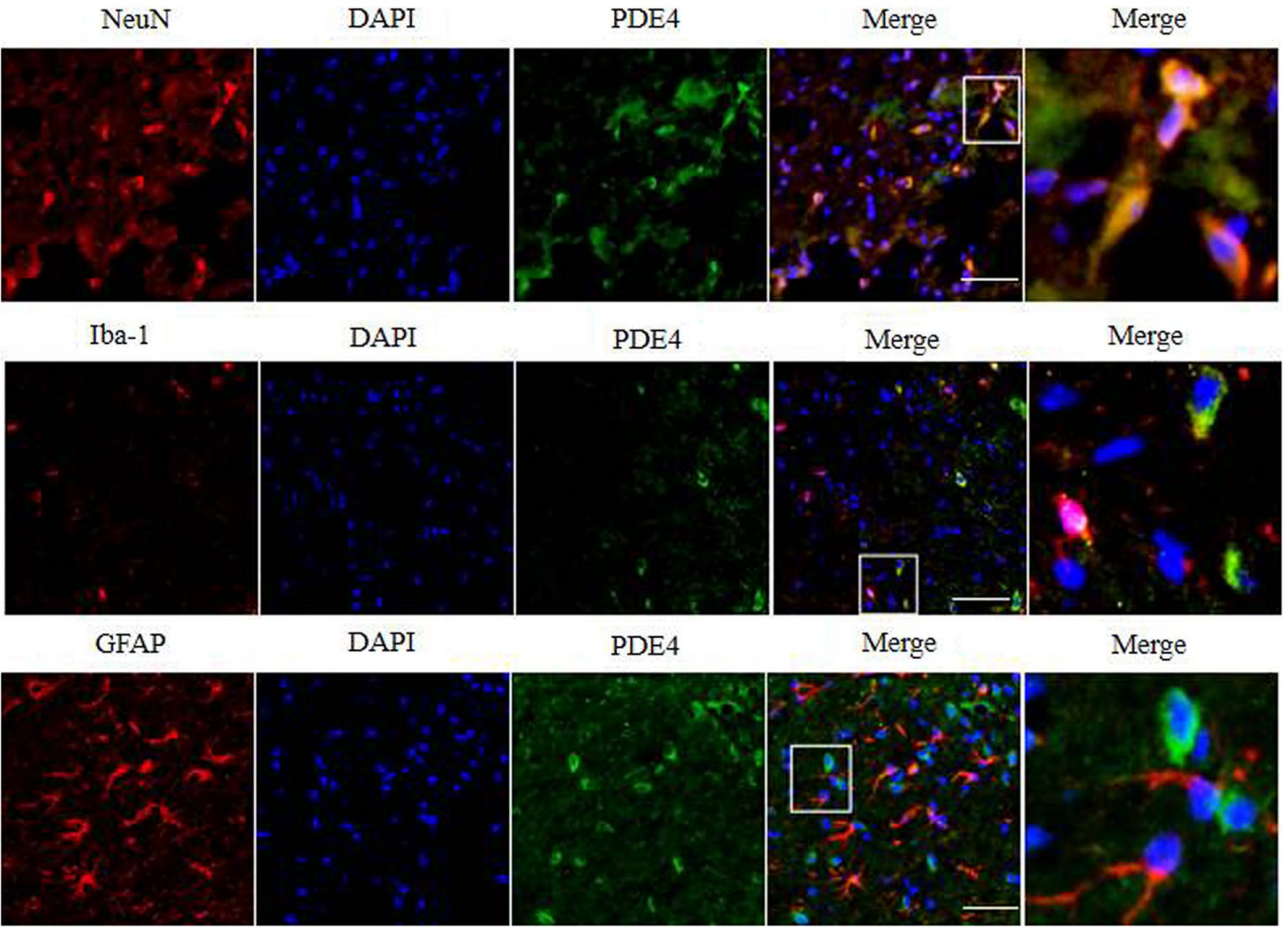
Increased PDE4 immunoreactivity around the haematoma on day 3 after ICH. PDE4 was predominantly expressed in neurons and was expressed at low levels in astrocytes and microglia. Scale bar = 20 µm.

The distribution of PDE4 was identified by double-immunofluorescence staining. As shown in Fig. 1, PDE4 was located predominately in neurons but not microglia or astrocytes around the haematoma on day 3 after ICH.

### The PDE4 inhibitor rolipram attenuates neurological dysfunction and brain oedema after ICH

**Figure 2:**
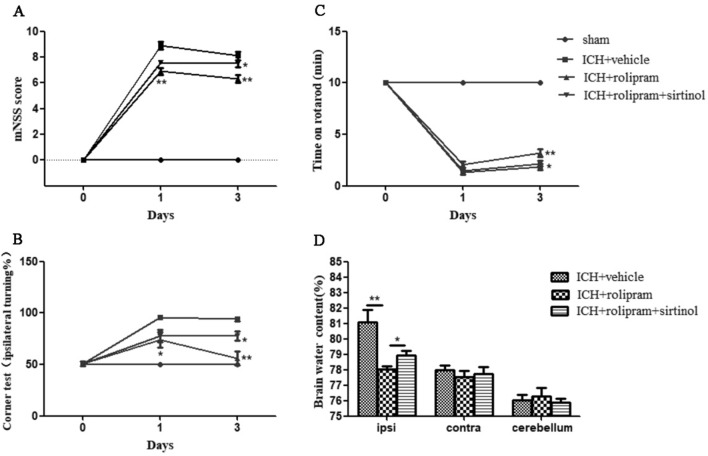
Rolipram ameliorated ICH-related motor deficits and reduced brain water content. (**A**–**C**) Behavioural performance was measured with mNSS, corner turning and time on the rotarod on day 1 and day 3 after ICH. The two-way ANOVA was applied for behavioral test. (**D**) Brain water content on day 3 after ICH. The Mann–Whitney *U* test was used to compare differences between groups in brain water content test. The data are presented as the means ± SEM. *p < 0.05, **p < 0.01.

To comprehensively determine the impact of rolipram on ICH in mice, neurobehavioural functions were evaluated at baseline and 1 and 3 days after ICH. The ICH score of the sham group was within the normal range, and the vehicle group exhibited higher neurological deficit scores than the sham group. These results indicated that ICH could impair neurological function in mice and that the ICH model was successfully established. The average scores of neurological deficits were significantly decreased after rolipram treatment. The administration of rolipram significantly attenuated the severity of behavioural symptoms on day 3 after ICH (*p* < 0.01 versus the ICH + vehicle group, Fig. 2A–C). Furthermore, brain water content was measured to evaluate the severity of brain oedema on day 3 after ICH. Brain water content was reduced by rolipram, with a more than 3% decrease (*p* < 0.05 versus the ICH + vehicle group, Fig. 2D).

The specific SIRT1 inhibitor sirtinol was used to verify the potential neuroprotective mechanism of rolipram against ICH. Sirtinol reversed the protective effect of rolipram on neurological dysfunction (*p* < 0.05 versus the ICH + rolipram group, Fig. 2A–C) and the reduction in brain oedema (*p* < 0.05 versus the ICH + rolipram group, Fig. 2D).

### Rolipram protects the permeability and integrity of the BBB

**Figure 3:**
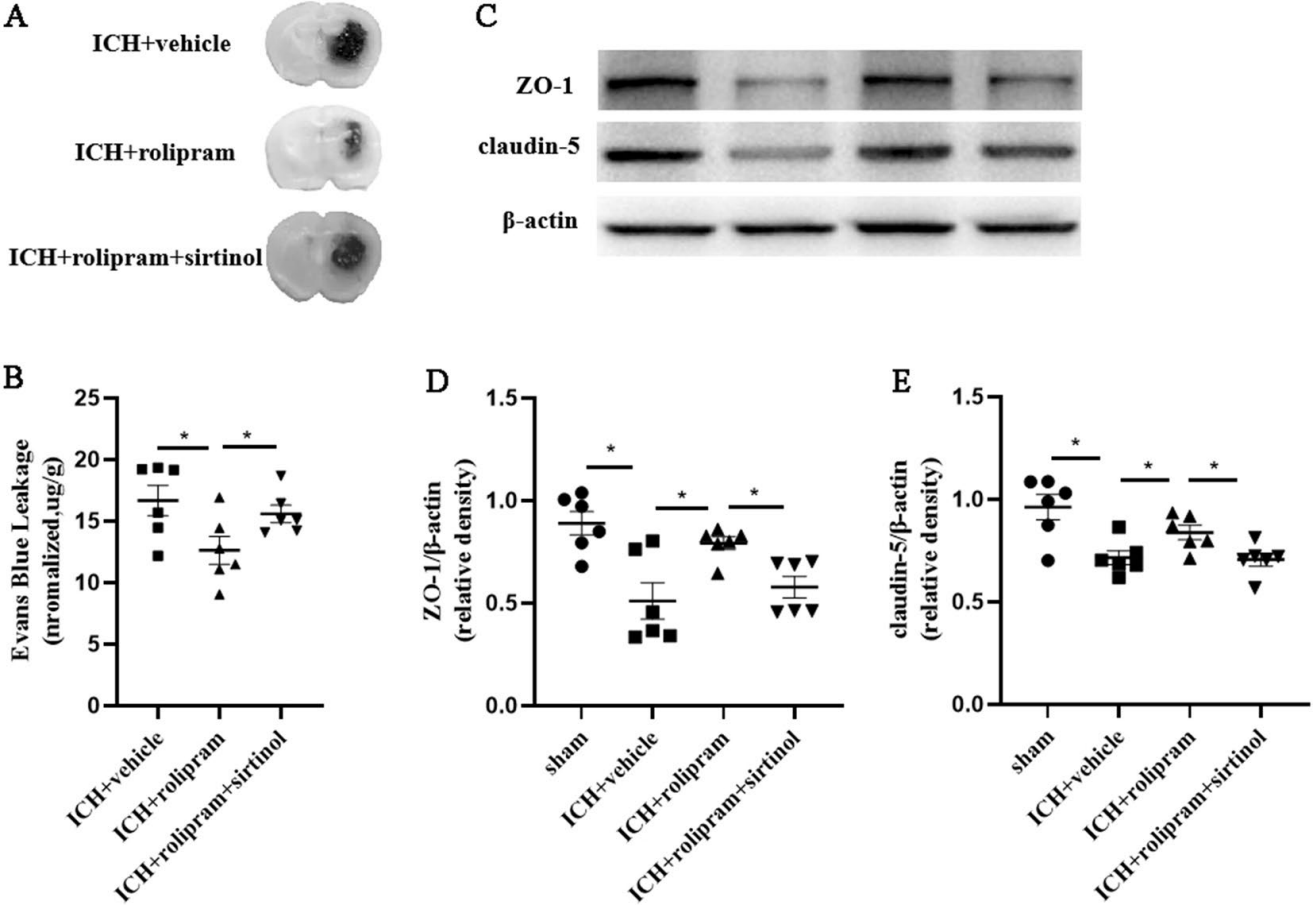
Rolipram alleviated BBB damage and EB leakage and increased the expression of tight junction proteins. (**A**,**B**) BBB permeability and EB leakage. (**C**–**E**) Western blot analysis of claudin-5 and ZO-1 in the different groups. The Mann–Whitney *U* test was used to compare differences between groups in biochemical and histological test. The data are presented as the means ± SEM. *p < 0.05 (Supplementary Information 1).

To determine the impact of rolipram on neurovascular function on day 3 after ICH, we examined the permeability of the BBB and the integrity of tight junctions in the brains of ICH mice. BBB leakage was examined and revealed that rolipram significantly reduced EB extravasation (*p* < 0.05 versus the ICH + vehicle group, Fig. 3A,B), and as expected, mice in the sirtinol group had significantly higher extravasation than those in ICH + rolipram group (*p* < 0.05 versus the ICH + rolipram group, Fig. 3A,B). Western blot analysis of tight junction proteins showed that the expression of claudin-5 and zonula occludens-1 (ZO-1) decreased after ICH (*p* < 0.05 versus the sham group, Fig. 3C–E) and was improved in the rolipram group compared with the vehicle group (*p* < 0.05, Fig. 3C–E). However, the use of sirtinol resulted in a decrease in tight junction protein expression (*p* < 0.05 versus the ICH + rolipram group, Fig. 3C–E).

### Rolipram alleviates neuronal apoptosis after ICH

**Figure 4:**
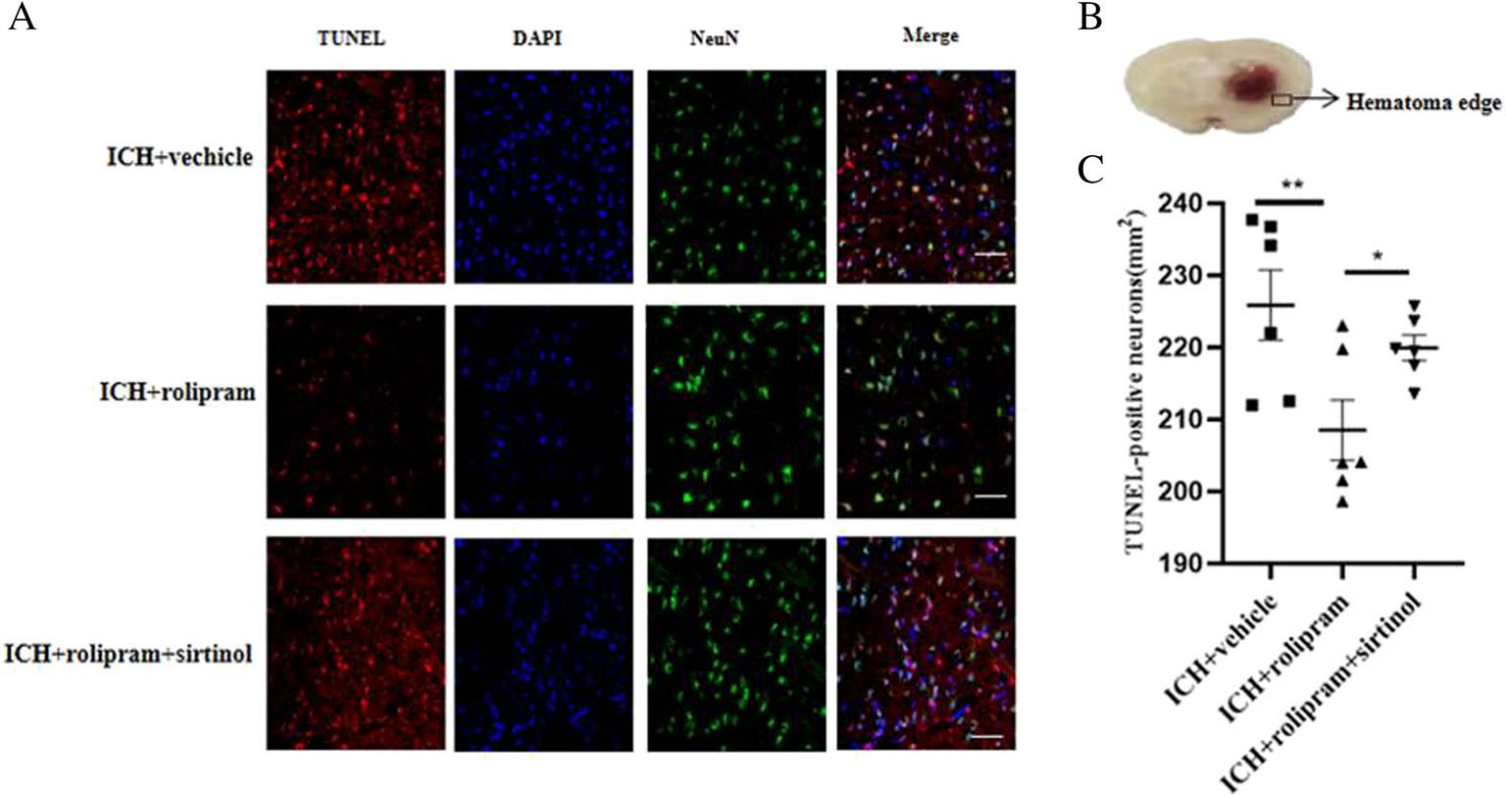
Rolipram attenuated neuronal apoptosis after ICH. (**A**) Representative TUNEL/NeuN photomicrographs in the different groups (scale bar = 20 µm). Fluorescence colours: TUNEL (red), DAPI (blue) and NeuN (green). (**B**) The box in the figure shows where apoptosis was measured. (**C**) Quantification of the numbers of TUNEL/NeuN-positive cells in the different groups. The Mann–Whitney *U* test was used to compare differences between groups in histological test. The data are presented as the means ± SEM. *p < 0.05, **p < 0.01.

Terminal deoxynucleotidyl transferase deoxyuridine triphosphate (dUTP) nick end labelling (TUNEL)/neuronal nuclei (NeuN) immunofluorescent double-labelling was conducted to further examine neuronal apoptosis on day 3 after ICH. The total numbers of TUNEL-positive neurons were significantly increased after ICH. Treatment with rolipram markedly reduced the number of TUNEL-positive neurons (*p* < 0.01, Fig. 4A–C), and this effect was reversed by sirtinol (*p* < 0.05, Fig. 4A–C).

### Rolipram ameliorates the inflammatory milieu

**Figure 5:**
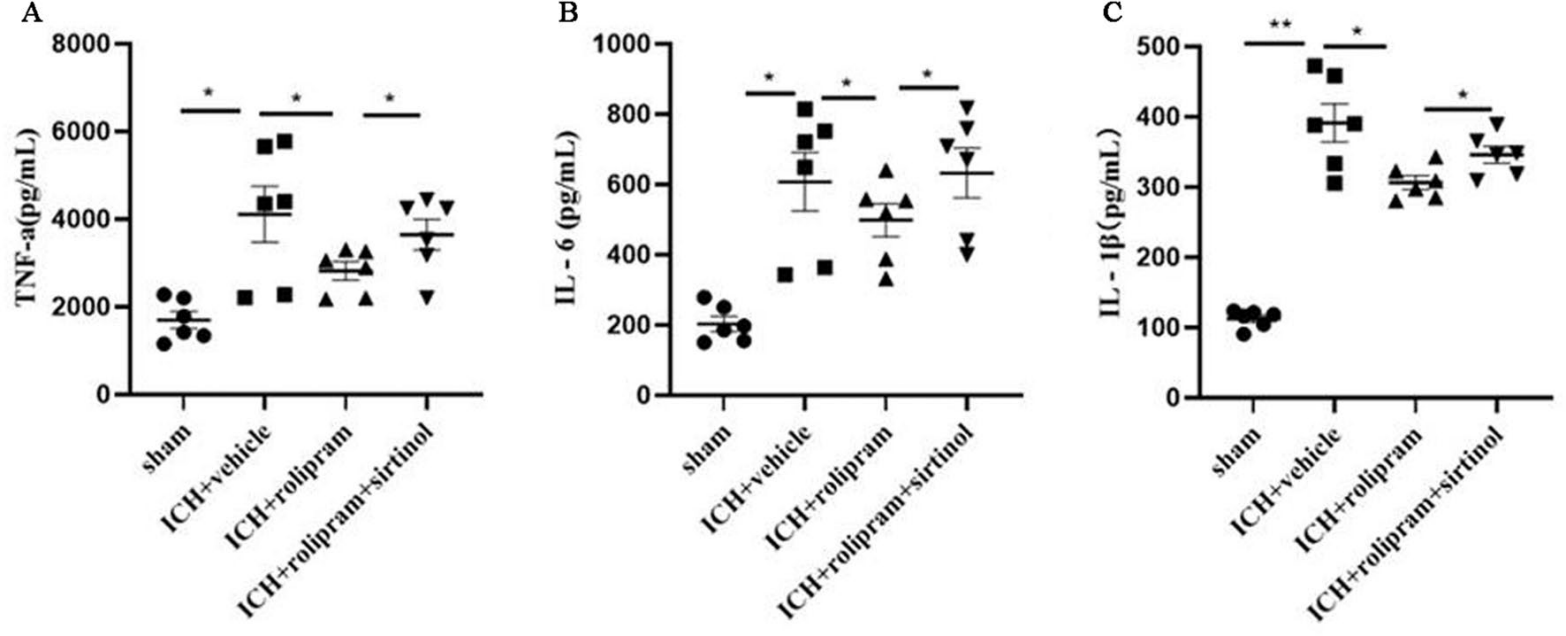
Rolipram alleviated the inflammatory environment in the brain after ICH. (**A**–**C**) TNF-α, IL-6, and IL-1β expression levels in the different groups. The Mann–Whitney *U* test was used to compare differences between groups in ELISA. The data are presented as the means ± SEM. *p < 0.05, **p < 0.01.

ICH significantly increased the expression of the inflammatory cytokines TNF-α, IL-6, and IL-1β in brain tissues around the haematoma on day 3 after ICH (*p* < 0.05, Fig. 5A–C). However, treatment with rolipram significantly reduced the production of these inflammatory factors compared to that in the vehicle group (*p* < 0.05, Fig. 5A–C). The opposite effect was observed in sirtinol-treated mice, which showed increased expression of TNF-α, IL-6, and IL-1β compared with that in the rolipram-treated group (*p* < 0.05, Fig. 5A–C).

### Rolipram-induced neuroprotection is mediated by the cAMP/AMPK/SIRT1 pathway

**Figure 6:**
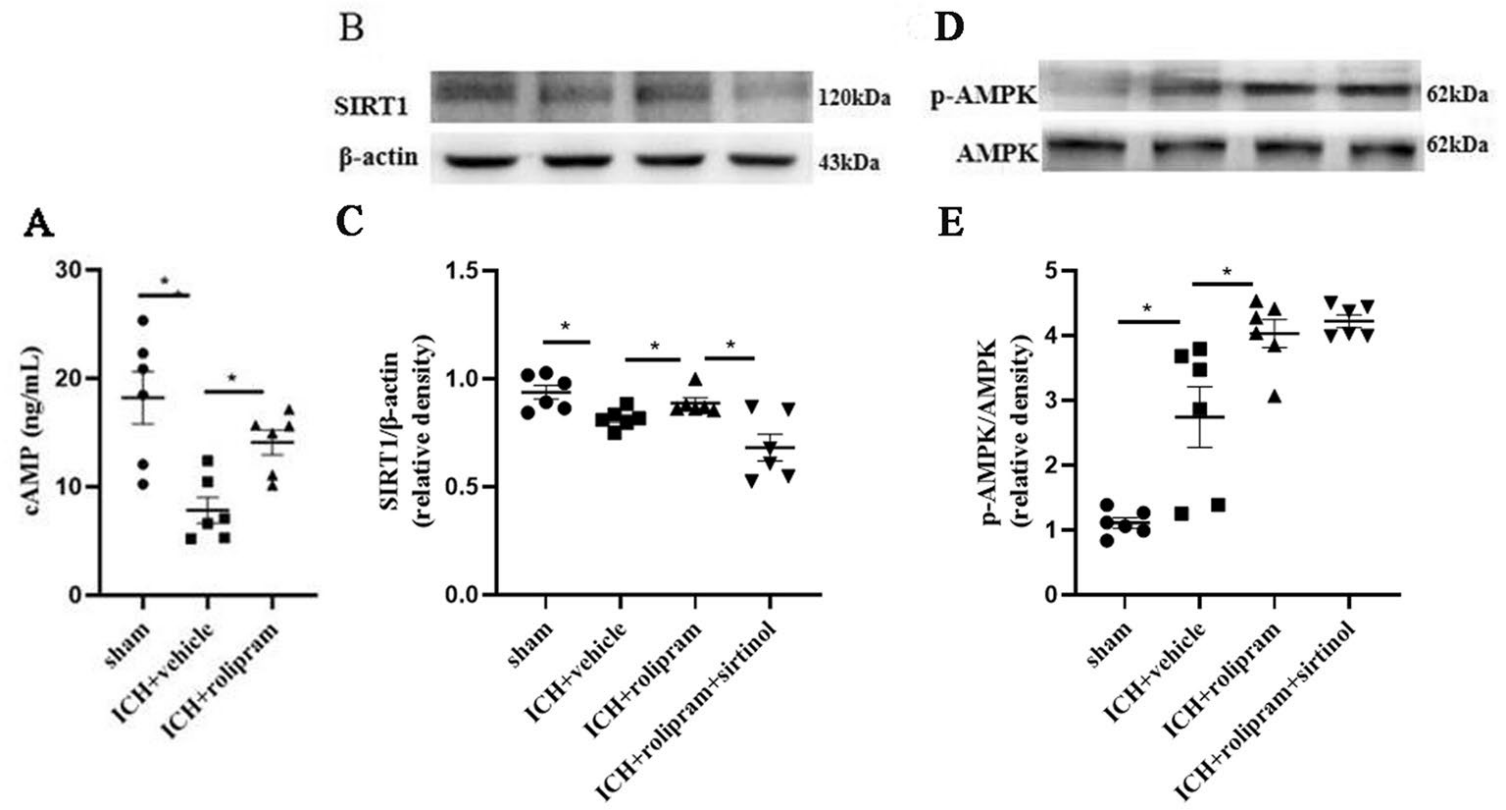
Rolipram activated the cAMP/AMPK/SIRT1 pathway after ICH. (**A**) The level of cAMP in the different groups. (**B**–**E**) Representative images and the quantification of western blot results showing SIRT1, p-AMPK and AMPK expression on day 3 after ICH. The Mann–Whitney *U* test was used to compare differences between groups in western blot. The data are presented as the means ± SEM. *p < 0.05 (Supplementary Information 2).

Three days after ICH surgery, brain tissues around the haematoma were prepared, and proteins were isolated to determine the activity in the cAMP/AMPK/SIRT1 pathway. We found that the expression levels of cAMP and SIRT1 were significantly decreased (*p* < 0.05, Fig. 6A–C), while the expression of p-AMPK was upregulated (*p* < 0.05, Fig. 6D,E) in the vehicle group. After rolipram treatment, cAMP, p-AMPK and SIRT1 expression levels were decreased significantly compared with those in the vehicle group (*p* < 0.05, Fig. 6A–E), suggesting that rolipram activated the cAMP/AMPK/SIRT1 signalling pathway. In addition, we observed that the expression of SIRT1 was inhibited in mice treated with the SIRT1-specific inhibitor sirtinol (*p* < 0.05, Fig. 6B,C). However, the effect of sirtinol on p-AMPK levels was not significant (*p* > 0.05, Fig. 6D,E).

## Discussion

In the current study, we investigated the potential protective effects of the PDE4 inhibitor rolipram after ICH and explored the possible biological mechanism. We made the following major observations: (1) On day 3 after ICH, PDE4 was located mainly in neurons according to visual reading of double-immunofluorescence staining; (2) rolipram exerted neuroprotection by alleviating neurological deficits, brain oedema, BBB destruction, neuronal apoptosis and inflammation; (3) rolipram significantly increased intracellular cAMP concentrations and upregulated the protein levels of SIRT1 and p-AMPK in the brain tissue surrounding the haematoma; and (4) these protective effect of rolipram could be blocked by sirtinol, a SIRT1 inhibitor. Our present findings indicated that the neuroprotective effect of rolipram on ICH was associated with the cAMP/AMPK/SIRT1 pathway and that PDE4 inhibition might be a potential therapeutic strategy for ICH treatment.

PDE4 is an active enzyme with an important physiological role that is widely distributed in the body and encoded by four homologous genes, PDE4A, B, C, and D. Except for PDE4C, the other three genes are widely expressed in the CNS^20^. A large number of studies have shown that PDE4 plays an important regulatory role in various types of tissue damage, such as acute kidney injury^21^, heart failure^22^ and asthma^23^. In addition, recent studies have shown that PDE4 is involved in the pathophysiological processes of various CNS diseases, such as ischaemic stroke^24^, experimental autoimmune encephalomyelitis (EAE)^25^ and Alzheimer’s disease(AD)^26^. Specifically, the overexpression of PDE4 further exacerbated the severity of these diseases^27,28^. Therefore, it is thought that PDE4 inhibitors might have potential therapeutic effects on a variety of neurological disorders^29^.

As a first-generation PDE4 inhibitor, rolipram has been widely investigated in preclinical studies of neurological diseases^30^. This compound is easily absorbed and can quickly penetrate the BBB to produce powerful anti-inflammatory and anti-apoptotic effects. Studies have shown that rolipram has a protective effect on CNS diseases. For instance, rolipram ameliorated cognitive dysfunction in a mouse model of AD by reducing neuronal damage^31^, and it has been proven to increase the phosphorylation of cAMP responsive element binding protein (CREB) and limit the expression of TNF-α and IL-10 in β-amyloid-induced cognitive impairment^32^. In addition, rolipram attenuated brain injury and BBB destruction by inhibiting neuroinflammation and reducing thrombosis in ischaemic stroke^33^, and it played a therapeutic role in the EAE model by stabilizing the BBB through anti-inflammatory effects^34^. In the present study, we first confirmed that PDE4 was mainly expressed on neurons on day 3 after ICH by double-immunofluorescence staining, indicating that PDE4 might participate in the pathological process of neuronal injury after ICH. Our results further suggested that rolipram may have a potential protective effect after ICH, in particular by improving neurological function scores, alleviating brain tissue water content and protecting BBB integrity.

A growing number of studies have confirmed that inflammation and apoptosis are two important factors associated with brain damage after ICH^35^. After ICH, neurological dysfunction can be exacerbated by an inflammatory cascade reaction via BBB disruption, brain oedema, and the initiation and amplification of the oxidative stress response, thereby increasing neuronal apoptosis and necrosis^36^. In accordance with a previous study^37^, our results showed that apoptotic neurons and inflammatory cytokine expression were significantly increased after ICH and could be partly reversed by rolipram treatment. These findings suggested that rolipram protected neurological function after ICH by maintaining the integrity of the BBB and alleviating neuronal apoptosis and inflammation.

PDE4 hydrolyses more than 80% of cAMP in neuronal tissues^38^ and mediates a series of complex biological processes by regulating intracellular cAMP concentrations^39^, including cell differentiation, energy metabolism, oxidative stress, immune response, and cell survival^40^. It was reported that cAMP/PKA pathway activation could induce endothelial autophagy by activating the downstream proteins AMPK and SIRT1^41^. Previous experiments have shown that in ageing-related metabolic diseases, rolipram could inhibit PDE4 and increase intracellular cAMP levels, leading to the activation of exchange protein activated by cAMP-1 (EPAC1) and its effector protein, which increases intracellular Ca^2+^ levels and ultimately activates the AMPK pathway and increases the activities of NAD+ and SIRT1^42^. Rolipram can also enhance autophagy in nerve cells in the primary spinal cord neuron M1 model by activating the cAMP/AMPK/SIRT1 pathway and alleviate nerve cell injuries^43^. It has been shown that SIRT1 activation could effectively reduce brain oedema, protect the BBB, block the progression of the inflammatory response and reduce apoptosis in CNS diseases^44,45^. In the present study, we found that rolipram increased the expression of p-AMPK and upregulated SIRT1 after ICH. Moreover, the upregulation of SIRT1 and neuroprotection induced by rolipram could be reversed by the SIRT1-specific inhibitor sirtinol. Previous findings indicated that the inhibition of PDEs provides neuroprotection by regulating the cAMP/AMPK/SIRT1 pathway in the ischemic brain^19^. Taken together with previous findings^19^, our observations suggested that the neuroprotective effect of rolipram was associated with the cAMP/AMPK/SIRT1 pathway. Previous studies have shown that PDE4 inhibitors are not direct activators of SIRT1 in ischaemic stroke but can stimulate SIRT1 by activating AMPK^16^. In this context, our results showed that the administration of sirtinol decreased SIRT1 protein expression, but the expression of p-AMPK was not affected, which is consistent with the above findings. We found that after ICH pAMPK was increased. However, the level of SIRT1 was decreased compared to that in sham group. We thought that there were several pathways regulating SIRT1 expression after ICH^19,46^. So we indicated pAMPK was increased in ICH without an increase in SIRT1. However, previous studies confirmed that rolipram exhibited greater emetic potency at doses between 0.01 and 0.1 mg/kg^47,48^. The clinical use of rolipram is limited because of its behavioural and emetic effects^49^. Therefore, newly derived selective, more potent and hopefully less toxic PDE-4 inhibitors could be more promising.

In conclusion, this study demonstrated the beneficial effects of rolipram on brain injury after ICH. The neuroprotective effects of rolipram included maintaining BBB integrity, reducing neuronal apoptosis and inhibiting the release of inflammatory cytokines, which might be mediated by activating the cAMP/AMPK/SIRT1 signalling pathway.

## Methods

### Animal preparation and study design

Healthy adult male C57BL/6 mice (6–8 weeks old) were purchased from Huafukang Biotechnology Co., Ltd. (Beijing, China). The mice were housed at 25 ± 1 °C in a humidity-controlled room at the animal care facility of Tianjin Medical University General Hospital (Tianjin, China) with 12 h light/12 h dark cycles and free access to food and water. Before the experiment, the mice were kept in separate cages for 1 week under the same conditions to adapt to the environment. Experimental protocols were approved by the Animal Experiments Ethical Committee of Tianjin Medical University General Hospital. All methods for animal study were carried out in accordance with relevant guidelines and regulations.

Then, the mice were randomly divided into four groups: sham group (n = 30), ICH + vehicle group (n = 30), ICH + rolipram (PDE4 inhibitor) group (n = 30) and ICH + rolipram + sirtinol (SIRT1 inhibitor) group (n = 30). Colocalization of PDE4 in nerve cells, neurological scores, brain water content, Evans blue (EB) leakage, neuronal apoptosis, inflammatory factor expression, and changes in key proteins in the cAMP/AMPK/SIRT1 signalling pathway were evaluated.

### Establishment of the ICH model

The ICH mouse model was established using collagenase injection^50^. In brief, the mice were anaesthetized by an intraperitoneal (i.p.) injection of 5% chloral hydrate (7 mL/kg) and fixed on the brain stereotaxic device. Then, the hair on the head was removed with ophthalmic scissors, and regional skin was cut approximately 1 cm in the middle of the head. The bregma point was taken as the origin, 0.5 mm forward and 2.3 mm to the right as the injection point. Then, an injection hole was drilled approximately 1 mm in depth, the pre-fixed micro syringe needle was inserted into the brain parenchyma depth of 3.7 mm, and 0.5 µL of normal saline containing 0.0375 U collagenase IV was injected into the brain parenchyma of 1 µL/min. Ten minutes after the injection was completed, the syringe was gradually removed in three steps, remaining in place for approximately 5 min at each step. Finally, the small hole was closed with bone wax, and the wound was sutured with surgical thread. The entire operation process was performed in an environment with a constant temperature room of 24 °C. After the operation, the mice were allowed free access to food and water. Sham-operated animals were subjected to the same surgical procedures, but no collagenase was injected. Neurological deficit evaluation was conducted 3 days after ICH according to Zea Longa's method^51^. Mice with Zea Longa scores of zero or four were excluded from the study.

### Drug administrations

According to previous studies, the PDE4 inhibitor rolipram (Selleck Chemicals, Houston, TX, USA) was dissolved in vehicle (0.5% DMSO in 1 mL of saline) to achieve a final concentration of 1 mg/mL^14^, and then 220–250 µL was injected intraperitoneally (equivalent to 10 mg/kg) immediately after ICH. Mice in the sham group and ICH + vehicle group received the same volume of sterile saline at the same time. Before establishing the ICH model, the SIRT1 inhibitor sirtinol (Selleck Chemicals, Houston, TX, USA) was diluted in vehicle (0.5% DMSO) to a concentration of 2 mmol/L^44^ and injected into one lateral ventricle. Intracerebroventricular injection was performed as previously described^52^. After being anaesthetized, the mice were fixed on the stereotactic apparatus in the prone position. The bregma point was taken as the origin, the injection point of the right lateral ventricle was determined to be 0.1 mm backward and 1 mm to the right of the bregma. A 10 µL micro injector was used, and the needle was inserted 2.0 mm below the skull into the right lateral ventricle. The injection rate was set as 1 µL/min. As described in a previous study, sirtinol was diluted in vehicle (0.5% DMSO) at a concentration of 2 mmol/L, and then sirtinol was injected in 3 µL^14^. The syringe remained in situ for an additional 10 min before removal. Then, the foramen was sealed with bone wax.

### Neurological scores

Ten mice were randomly selected for behavioral study. The modified neurological severity score (mNSS), corner turning test and rotarod test were used to evaluate neurological function, including motor ability and sensory, reflex and balance functions, at baseline and at 1 and 3 days after ICH. The neurological scores were evaluated by two investigators who were blind to the treatments/grouping.

### Brain water content

Six mice were used for brain water content. Mice were sacrificed under deep anaesthesia on day 3 after ICH. The whole brain was immediately removed, and each hemisphere was weighed separately. Then, the brain was dried in an oven at 100 °C for 24 h to measure the dry weight. The brain tissue water content (%) was calculated as follows: (wet weight-dry weight)/wet weight × 100%.

### EB staining

#### Brain tissue sections

BBB permeability was investigated on day 3 after ICH by measuring EB leakage. There were 6 mice used for EB staining. EB dye (2%; 2 mL/kg, Solarbio) was injected into the tail vein 2 h before sacrifice. Then, the mice were perfused with phosphate-buffered saline (PBS) (pH 7.4, 4 °C) through the left ventricle. Brain tissue was collected and placed in 4% paraformaldehyde for fixation overnight. The fixed brain tissue was placed in a mouse brain mould, cut into brain slices at a thickness of approximately 1 mm along the coronal position and placed on glass slides to observe the exudation of EB on the side of the cerebral haemorrhage. The quantitative detection of EB extravasation was conducted in the whole surgical hemisphere.

#### EB content determination

After heart perfusion, the surgical cerebral hemisphere was collected and weighed, and then the brain tissue was cut into pieces and ground into homogenate. Brain tissue homogenate was resuspended in 5 mL of dimethylformamide solution. Then, after 72 h of incubation at 60 °C and centrifugation at 1000 rpm for 5 min, the supernatant was collected for analysis. The absorbance value at 570 nm was measured by a microplate reader. The concentration of the standard solution was preconfigured, and the standard curve was drawn according to the absorbance value of the standard substance. Then, the amount of EB per gram of brain tissue was calculated according to the formula. EB content (µg/g) in brain tissue was calculated as follows: EB concentration (µg/mL) × dimethylformamide volume (mL)/brain wet mass (g).

### TUNEL staining

TUNEL staining was performed to detect neuronal damage according to the manufacturer’s instructions (Roche, USA). Six mice were used for TUNEL staining. Brain tissue surrounding the haematoma was used to measure neuronal damage by TUNEL staining. The extent of neuronal damage was evaluated by calculating the average number of TUNEL-positive neurons in six sections randomly. Apoptotic cells in the brain tissue surrounding the haematoma showed red fluorescence in the nucleus. The sections were visualized by a fluorescence microscope. TUNEL-positive cells were assessed with Image J software. The results were calculated as the average number of TUNEL-positive neurons and are expressed as the number of positive cells/mm^2^ tissue.

### Immunofluorescence staining

After heart perfusion, the brains were immersed in 30% sucrose overnight at 4 °C until they sank to the bottom of the centrifuge tubes. Coronal sections (8 µm) were prepared using a freezing slicer, and the sections were stored at − 20 °C until staining. For immunostaining, the sections were blocked in 10% foetal bovine serum supplemented with 0.3% Triton X-100 for 1 h at room temperature. Next, the sections were incubated with a combination of primary antibodies at 4 °C overnight in a black wet box. Primary antibodies included rabbit anti-PDE4 (1:200, ab14628, Abcam, USA), mouse anti-neuron (NeuN) (1:500, ab104224, Abcam, USA), goat anti-ionized calcium binding adaptor molecule 1 (Iba-1) (1:200, ab076, Abcam, USA), and goat anti-glial fibrillary acidic protein (GFAP) (1:200, ab53554, Abcam, USA). Brain tissue surrounding the haematoma was used to measure the expression of PDE4, NeuN, Iba-1, and GFAP. Sections were washed three times in PBS before being incubated with secondary antibodies for 1 h at room temperature. Images were taken using a fluorescence microscope (Olympus PX51, Olympus Corporation). The PDE4 localization in nerve cells was observed.

### Western blotting

There were 6 mice conducted western blotting. Brain tissue surrounding the haematoma was homogenized by sonication in radioimmunoprecipitation assay (RIPA) buffer containing protease and phosphatase inhibitors (Roche Diagnostics) and centrifuged at 12,000*g* for 20 min. Proteins were resolved by 10% SDS-PAGE and transferred onto polyvinylidene difluoride (PVDF) membranes. After being blocked with 5% skim milk in Tris-buffered saline with Tween-20 (TBST), the membranes were incubated with primary antibodies (rabbit anti-claudin-5, 1:1000, Abcam; rat anti-ZO-1, 1:1000, Abcam; rabbit anti-SIRT1, 1:1000, Cell Signaling Technology; rabbit anti-phospho-AMPK(Ser 485), 1:1000, Cell Signaling Technology; rabbit anti-p-AMPK, 1:1000, Cell Signaling Technology; mouse anti-β-actin, 1:2000, Zhongshan Jinqiao, Beijing) overnight at 4 °C. Subsequently, the PVDF membranes were washed three times with TBST and incubated with secondary antibodies (1:5000, Zhongshan Jinqiao, Beijing) at room temperature for 60 min. The blots were visualized using enhanced chemiluminescence (ECL, Cell Signaling Technology) reagents, and band intensities were assessed with Image J software.

### Enzyme-linked immunosorbent assay (ELISA)

There were 6 mice in ELISA test. Brain tissue samples were collected from the area surrounding the haematoma. Tissue homogenates were prepared with PBS and phenylmethylsulfonyl fluoride. The levels of cAMP, TNF-α, IL-6, and IL-1β were quantified using ELISA kits (Elabscience Biotechnology Co., Ltd, Wuhan, China) according to the manufacturer’s instructions.

### Statistical analysis

Statistical analyses were performed using GraphPad Prism software. Differences were considered significant at *p* < 0.05. The Mann–Whitney *U* test was used to compare differences between groups in the biochemical and histological tests. Two-way ANOVA was applied for behavioural tests. The data are expressed as the means ± SEM.

### ARRIVE guidelines statement

The study was carried out in compliance with the ARRIVE guidelines.

## Supplementary Information

Supplementary Information 1.

Supplementary Information 2.

Supplementary Legends.
