# Supplementary figures and images for: The protective effect of the PDE-4 inhibitor rolipram on intracerebral haemorrhage is associated with the cAMP/AMPK/SIRT1 pathway
Source: Sci Rep. 2021 Oct 5;11:19737. doi: 10.1038/s41598-021-98743-w (PMC8492710; doi:10.1038/s41598-021-98743-w)

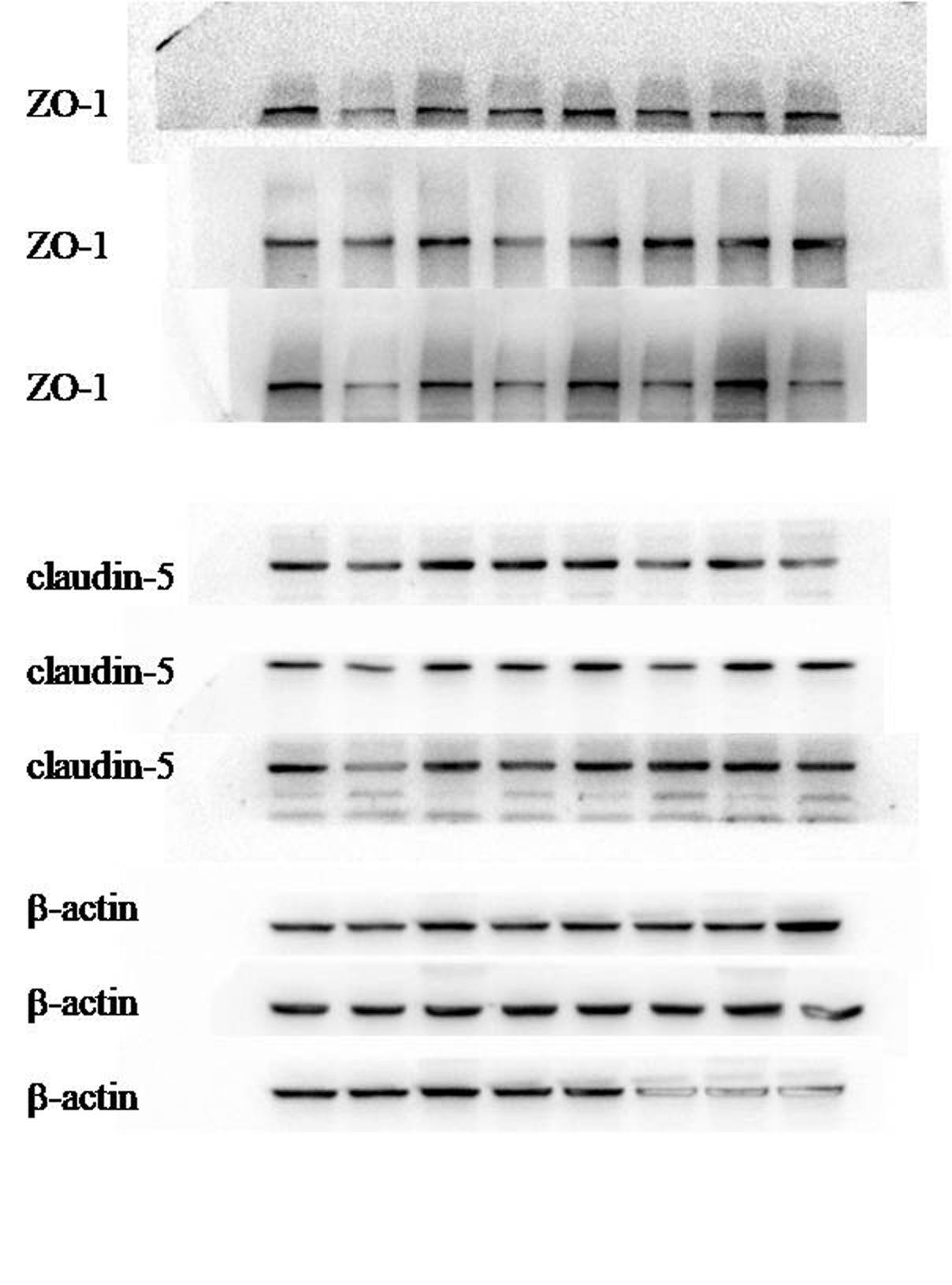

Supplement: Supplementary file 1 — Supplementary Information 1. [file 41598_2021_98743_MOESM1_ESM.jpg]

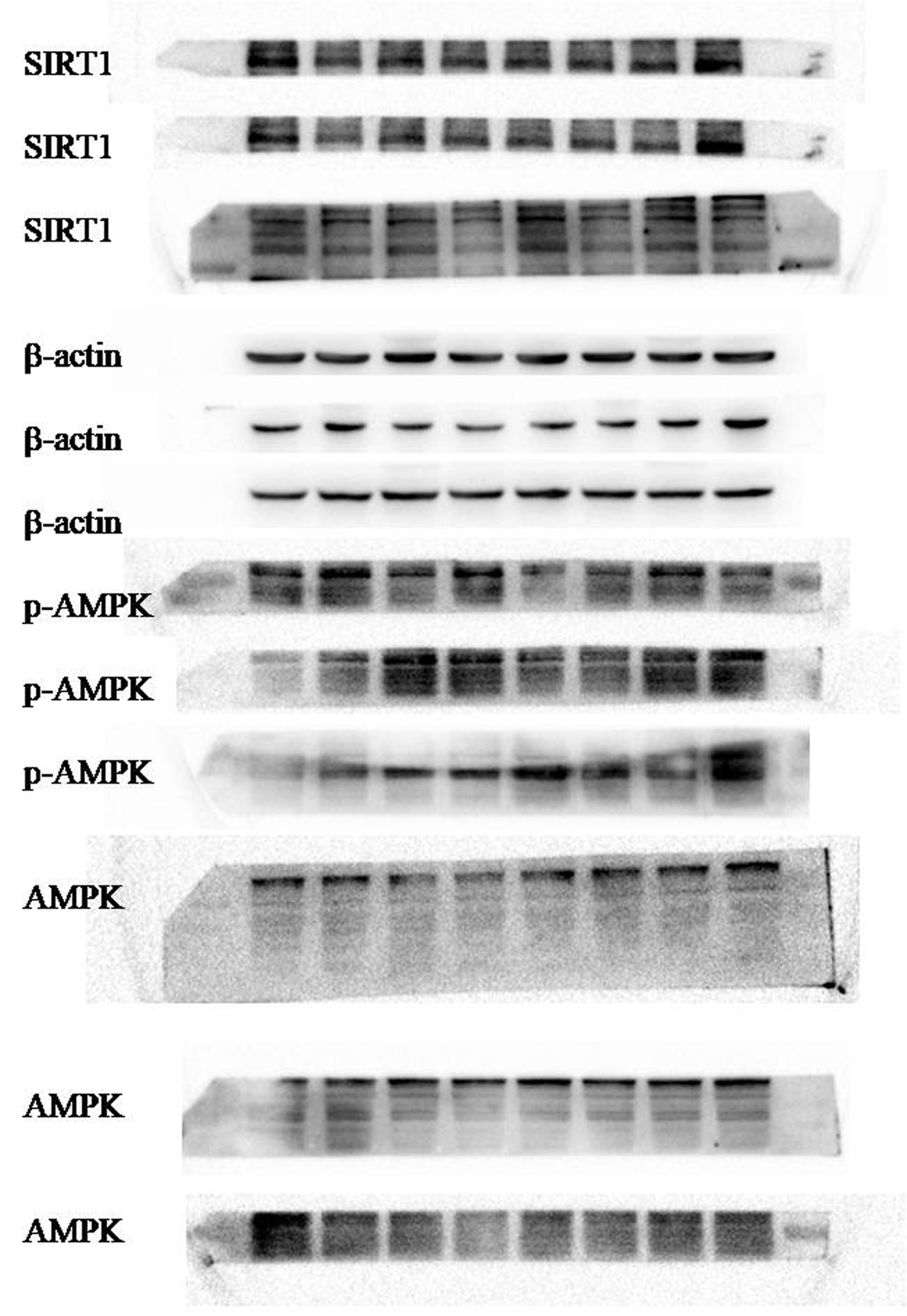

Supplement: Supplementary file 2 — Supplementary Information 2. [file 41598_2021_98743_MOESM2_ESM.jpg]
