# Supplementary material for: The protective effect of the PDE-4 inhibitor rolipram on intracerebral haemorrhage is associated with the cAMP/AMPK/SIRT1 pathway
Source: Sci Rep. 2021 Oct 5;11:19737. doi: 10.1038/s41598-021-98743-w (PMC8492710; doi:10.1038/s41598-021-98743-w)
Supplement: Supplementary file 3 — Supplementary Legends. [file 41598_2021_98743_MOESM3_ESM.docx]

### Supplementary material

### Supplementary material 1 Original versions of Fig. 3

### Supplementary material 2 Original versions of Fig. 6
